# Role of Dendritic Epidermal T Cells in Cutaneous Carcinoma

**DOI:** 10.3389/fimmu.2020.01266

**Published:** 2020-07-14

**Authors:** Jian Xiang, Minghui Qiu, Hongyi Zhang

**Affiliations:** Department of Microbiology and Immunology, School of Medicine, Jinan University, Guangzhou, China

**Keywords:** dendritic epidermal T cells, γδ T cells, epidermis, squamous cell carcinoma, melanoma

## Abstract

Dendritic epidermal T cells (DETCs) are γδ T cells expressing invariant Vγ5Vδ1 T cell receptor (TCR) in murine epidermis. Initially, the development and the maturation of DETC progenitors are mediated by skint-1, TCR, and cytokines in the fetal thymus. Then, the DETC progenitors migrate to the epidermis with the guidance of selectins, CCR10, CCR4, *etc*. Eventually, mature DETCs proliferate and maintain a homeostatic population in the epidermis through IL-15 and aryl hydro-carbon receptor signaling. In “stressed” skin, DETCs are activated, exhibiting features such as a round morphology, cytotoxicity, and production of cytokines. In cutaneous carcinoma, DETCs generally inhibit tumor development directly in non-major histocompatibility complex-restricted manner, with the assistance of cytokines. DETCs also recognize and inhibit tumor via TCR, non-TCR receptors (such as 2B4 and NKG2D), or both. This study summarizes the biogenesis and the function of DETCs in cutaneous carcinoma and clarifies the essential surveillance role in the epidermis that DETCs play. As there are no DETCs in human epidermis but only human epidermis γδ T cells, we need to understand the anti-tumor pathways used by DETCs to find analogous immune pathways in human skin, which could be exploited for novel therapeutics.

## Introduction

The γδ T cells are abundant in epithelial surfaces of the skin, intestine, lung, *etc*. (1). The skin is comprised of the epidermis, the basement membrane, and the dermis. The epidermis consists of 95% keratinocytes and 5% immune cells, including Langerhans cells and T cells that are predominantly epidermal γδ T cells (2, 3). The dermis contains dermal γδ T cells and variant immune cells, including αβ T cells, macrophages, dendritic cells, *etc*. The epidermal γδ T cells are different from the dermal γδ T cells in the T cell receptor (TCR) chains and shapes. In mouse, compared with the round dermal γδ T cells expressing Vγ4, Vγ2 but not Vγ5 TCR (4), the epidermal γδ T cells are dendritic and exclusively express Vγ5 TCR, therefore termed as dendritic epidermal γδ T cells (DETCs). This dendritic morphology of DETCs may be localization specific as the skin-resident memory CD8^+^ T cells are also dendritic in the epidermis (3, 5). The dendritic morphology of DETCs may be shaped by CD103 and E-cadherin (6, 7).

DETCs are unique in rodents, and similar γδ T cells reside in the epidermis of some species (8), and DETCs are reported to play an important role in wound healing and surveillance on tumors (8). In rat epidermis, the majority of T cells are dendritic γδ T cells, with Vγ and Vδ chains highly similar to DETCs (9). In cattle epidermis, the skin-resident γδ T cells are still dendritic but with different Vγ chain and Vδ chain compared with DETCs (10). In humans, the γδ T cells equivalent to DETCs are to be discovered; only a small subset of γδ T cells expressing the Vδ1 TCR reside in the epidermis, termed as human epidermal γδ T cells. The human epidermal γδ T cells also promote wound healing by secreting insulin-like growth factor 1 and are also cytotoxic to cutaneous carcinoma as DETCs (11–13). However, the human epidermal γδ T cells are still different from DETCs in terms of the molecular mechanisms of homing to the epidermis, activation, and antigen recognition (14, 15), and human epidermal γδ T cells are round in morphology instead of dendritic. In this study, we summarize the biogenesis of DETCs and their function roles in cutaneous carcinoma and hope that these mechanisms can provide cues to the study of human epidermal γδ T cells in parallel.

## Biogenesis of DETC

DETCs are derived from DETC progenitors that are the first T cells generated in the thymus at embryonic day 13 (8). A few mechanisms are reported about the development and the maturation of the DETC progenitors. Skint-1, a member of the butyrophilin-like (Btnl) family proteins derived from mature thymic epithelial cells with activated rank signaling (16), is identified as the key molecule in promoting the selective development of Vγ5^+^ DETC progenitors (17). Skint-1 determines the differentiation direction of fetal thymocytes through a CDR3-like loop-dependent manner (17). After receiving the Skint-1 signal, the DETC progenitors provoke differentiation and produce IFN-γ by activating the Egr3-mediated pathway while suppressing Sox13 and RORγt that are essential for other γδ T cells that produce IL-17 (18). Although Skint-1 is not a γδTCR ligand, the Skint-1-mediated selection might be through a TCR-related manner because Egr3, Sox13, and Rorc are downstream molecules of TCR signaling (18). TCR–ligands interaction is also essential for the maturation of DETC progenitors. TCRs induce the expression of sphingosine-1-phosphate receptor 1 in DETC progenitors (19). The downstream of TCR signaling in mice only have a delayed DETC accumulation but not any effect on the DETC compartment in the epidermis (20). Therefore, the TCR–ligands signaling might only regulate the development of DETC progenitors in the thymus. Besides the cell–cell communication, the cytokines derived from fetal thymocytes promote the development of DETC precursors. IL-7 and IL-7R signaling is essential for TCR gene transcription in a JAK/STAT pathway-dependent manner (21). IL-2 and IL-15 promote the survival of DETC precursors (21).

DETCs are located in the basal layer of the epidermis. Therefore, DETC precursors need to migrate from the thymus to the epidermis via the following steps: (1) adhering to the endothelial capillary in the dermis and (2) extravasation and locating to the epidermis (22). For the first step, DETC precursors express ligands to bind to the selectins expressed on the vascular endothelium. Although the exact ligands have not been identified, evidences show that DETCs are dramatically reduced in mice lacking E-selectins and P-selectins (23). For the second step, the DETC precursors express high levels of CC-chemokine receptor 10 (CCR10), which is the receptor of CC-chemokine ligand 27 expressed by keratinocytes (24). DETCs are markedly reduced in mice lacking CCR10 because the DETC precursors are halted in the dermis (25). A small subset of DETC precursors is homing to the epidermis in a CCR4-dependent manner (23). The Vγ5 TCRs might be important for the DETC precursor migration and epidermal localization (20); however, TCR is also reported to be not specific for DETC migration and homing to the epidermis. Further investigations are needed (26–28).

Once the DETCs home in the epidermis, they proliferate exponentially along with the growth of the skin after birth in an IL-15-dependent manner as DETCs are decreased in IL-15- or IL-15R-deficient mice, while IL-15 is secreted by keratinocytes (29). In adults, DETCs are not supplied by circulating γδ T cells from hematopoietic stem cell but keep a homeostatic number by self-renewal in an aryl hydro-carbon receptor (AHR)-dependent manner. The AHRs are activated by ligands from the DETC cytoplasm. When lacking the AHR signaling, the DETCs cannot proliferate after homing in the epidermis (30). DETCs also produce insulin-like growth factors (IGFs) to prevent themselves from apoptosis (13). The Vγ5 TCR is important for the homeostatic maintenance of mature DETCs in adults (31). Therefore, after homing to the epidermis, the mature DETCs proliferate and maintain a homeostatic population.

DETCs need to be activated to play a functional role in damaged skin and cancer. In steady-state skin, DETCs extend their dendrites to the suprabasal layers and closely contact with keratinocytes (32). In pathological-state skin, activated DETCs become motile by losing the dendrites (32). DETCs may be activated by co-culturing with transformed keratinocytes and protect keratinocytes from apoptosis in an IGF1-dependent manner (13, 33). The TCRs are essential for DETC activation by recognizing antigens from keratinocyte or Langerhans cells (34, 35). Damaged or stressed keratinocytes express TCR ligands that can activate DETCs in a non-major histocompatibility complex (MHC)-restricted manner (35–37). Beyond TCR, the complete activation of DETCs requires co-stimulatory signals such as junctional adhesion molecule-like (JAML) (38), CD100 (39), 2B4 (40), and natural killer group 2D (NKG2D) (41). JAML expressed in DETC is similar to CD28/B7 in αβ T cell (38). CD100 expressed in DETC is a receptor for plexin B2-mediated signaling in keratinocyte to initiate DETC activation, with a morphology change (39). 2B4 expressed in DETC is associated with tumor target recognition (40). NKG2D expressed in DETC is a receptor for stress-induced proteins to activate DETC in responding to tumor or cutaneous wound (42, 43). The cytokines are also important for DETC activation. DETCs freshly isolated from skin can be activated by IL-2 (40), and activated DETCs produce IL-2 (44). IL-7 and IL-15 from keratinocytes and fibroblast activate DETCs (45–47). In contrast, the activation of DETCs is inhibited by the E-cadherin of keratinocytes (7). The DETC expression of JAML (38), CD100 (39), and NKG2D (43) are critical for wound healing. The DETC expression of 2B4 (40), NKG2D (41, 42), and IL-2 (44) may facilitate the cytotoxic potential to tumor cells.

## Role of DETC in Cutaneous Carcinoma

A total of 90% of cutaneous carcinomas are comprised of basal cell carcinoma (BCC), squamous cell carcinoma (SCC), and melanoma. In general, the inhibition of cutaneous carcinoma by activated DETCs relies on three consecutive signals: TCR in MHC—restriction independent (15), non-TCR receptors such as NKG2D (48), 2B4 (40), or cytokines such as IL-2 (44), and IFNγ (38, 48, 49).

### DETC in Non-melanoma Skin Cancer

BCC and SCC are usually categorized as non-melanoma skin cancer (50). BCC is the most common skin cancer, which starts from the base cell layer of the epidermis. SCC is the second common cutaneous carcinoma from damaged keratinocytes (50, 51). The non-melanoma skin cancer may be caused by solar UV radiation or chemicals such as arsenic (52, 53).

Majority of BCCs and 50% of SCCs are caused by solar UV radiation. In chronic UV radiation, the DNA repairing mechanism caused gene mutation and genome instability, which are responses for tumor formation. PTCH1 and P53 mutations drive BCC and SCC initiation, respectively (54, 55). DETC is the major antitumor player in murine epidermis. DETC can directly lyse the SCC cell line Pam 212 monolayer effectively (56) or inhibit the tumor cells by inducing CD8+T cells (57). DETC can lyse the PDV tumorigenic keratinocyte cell line (42) but not the normal keratinocyte cells *in vitro* (56). Therefore, the DETC's cytolytic activity may be tumor cell specific. The DETCs protect the keratinocyte from UV-caused DNA damage by reducing γH2AX, a cyclobutane pyrimidine dimer. UV-damaged keratinocytes secrete IL-1β, which triggers DETCs to produce IL-17A, and in turn, IL-17A upregulates molecules linked to DNA repair response and limits γH2AX expression in keratinocyte cells (58). The DETC population is decreased in UV-irradiated epidermis (57). Therefore, DETCs might have a potential role in preventing UV-induced skin cancer, and further studies are needed. However, IL-17A plays a dual role in promoting both tumor growth and antitumor immunity in skin cancer. On one hand, IL-17A accelerates the proliferation of skin epithelial cells to promote tumorigenesis (59, 60). IL-17A also promotes the tumor microenvironment formation by attracting an infiltration of immune cells (61). In murine models of ovarian cancer and pancreatic ductal adenocarcinoma, the IL-17-producing γδ T cells (not DETCs) are proliferative, active, and may directly inhibit adaptive antitumor immunity by producing PD-L1 and Galectin-9 (62, 63). Whether the tumor-infiltrating immune cells together with DETCs can promote tumorigenesis and tumor progression needs to be investigated. On the other hand, the IL-17-producing CD8^+^ T induces tumor regression in mice with vascularized B16 melanoma (64). The IL-17-producing γδ T cells enhance chemotherapy to mice with fibrosarcoma (65). Th17 cells activate endogenous cytotoxic CD8^+^ T cells, leading to tumor regression in melanoma (66). The generation of IL-17-producing T cells with different phenotypes in response to variant tumor contexts would explain the conflicting observations. Whether IL-17 plays a role in DETC-mediated antitumor immunity needs to be studied. In an UV-induced SCC model, DETCs can inhibit the activation of CD4^+^T cells, but not CD8^+^T cells, within 3 days after UV radiation, resulting in an accelerated tumor growth (67).

Aside from UV, SCC may also be induced by chemicals. In a 7,12-dimethylbenz(a)anthracene (DMBA)/12-O-tetradecanoylphorbol-13-acetate (TPA)-induced SCC model, the DETCs show an anti-tumor role as γδ T-depleted mice are more acceptable for tumors than the wild-type mice. DETCs eliminate DMBA/TPA-induced SCC by expressing IFNγ and NKG2D, therefore promoting the therapeutic effect of rapamycin on SCC (68). IFNγ promotes the migration, activation, and cytotoxicity of DETCs in SCC. NKG2D, a receptor of natural killer cells, is only expressed in DETC in murine epidermis. The expression of ligands for NAG2D, such as Rae-1 and H60, is inducible in SCC by DMBA/TPA treatment (42). Blocking NKG2D can inhibit DETC activation, but whether these ligands activate DETCs directly or indirectly needs more investigation. Rae-1 can activate DETCs directly without TCR signaling (69, 70). H60c can directly activate DETCs to produce IL-13 (71), but H60c is also reported to only provide co-stimulatory signals for DETC activation, failing to activate DETCs directly (69, 72). Thus, DETCs eliminate tumor mediated by NKG2D, but the NKG2D signaling of DETCs may response differently in a different stimulation content.

Cutaneous lymphoma, a rare subtype of non-Hodgkin lymphoma, starts from the lymphocytes in the skin but is not classified as cutaneous carcinoma. As for the well-established non-Hodgkin lymphoma cell line YAC, DETCs directly kill YAC cells by producing perforin and granzymes (56), and this cytotoxicity is not MHC-restricted (73, 74). The anti-tumor potential of DETCs is strengthened in the presence of cytokines. 2B4, initially found in NK cells and T cells, associate with non-MHC-restricted recognition to tumor targets (75, 76), is expressed in DETC, and mediates the killing of tumor cells by DETC (40). IL-2 enhances the cytotoxicity of DETC to lymphoma cells by stimulating 2B4 expression (40). The DETCs activated by CoA produce IL-2, which stimulates DETCs to kill YAC cells (40, 44). IL-7, produced by keratinocytes, is also critical for DETC activation to acquire a cytotoxic capability to lymphomas (56). The cytotoxicity of DETC directly to lymphoma cells can be strengthened by cytokines.

### DETC in Melanoma

Melanoma starts from melanocytes and is very aggressive and metastatic. Melanoma occupies 1% of cutaneous carcinoma cases but is the most lethal event in a cutaneous carcinoma patient. DETC cell line AU16 inhibits melanoma progression *in vivo* and kills melanoma cells in cytotoxicity *in vitro* (77). The DETC cell line AU16, derived from C3H mice, is an IL-2-dependent cell line and cytotoxic to melanoma cell lines and chemo-induced fibrosarcoma *in vitro* (77). The injection of mixed AU16 cells and melanoma cells can delay the melanoma growth *in vivo* (77). In another study, the inhibition of DETCs to melanoma may be tumor specific as normal keratinocytes are not affected (56). The inhibition of DETCs on melanoma is IL-2 dependent and needs a close contact between DETCs and melanoma (56). Microscopically, DETCs destruct melanoma monolayers by adhering to tumor cells first and then gradually forming enlarged discrete foci to disrupt the melanoma cells. DETCs may inhibit melanoma in a NKG2D-dependent manner as NKG2D ligands are largely expressed in melanoma (78). The studies of DETCs on inhibiting melanoma are limited, and further investigations are needed.

## Conclusion and Perspective

DETCs are the resident γδ T cells, with a dendritic morphology, in murine epidermis. Once the skin is damaged or has tumor, DETCs are activated by cytokines (such as IL-17, IL-15, and IL-2) or signaling directly from keratinocytes and Langerhans cells. The activated DETCs generally inhibit tumor progress but also promote tumor development in a certain tumor microenvironment. However, our understanding of the biology of DETCs is still largely limited, particularly in the area of DETCs responding to a skin tumor microenvironment. How do the DETCs maintain hemostasis in a skin tumor microenvironment? How do the DETCs communicate with the tumor-infiltrated immune cells and with the neighbor cells in the epidermis? We need to understand the anti-tumor pathways used by DETCs to find analogous immune pathways in human skin which could be exploited for novel therapeutics.

## Author Contributions

JX participated in the design of this study and organized the manuscript. MQ wrote the draft of the manuscript. HZ designed and revised the manuscript. All authors contributed to the article and approved the submitted version.

## Conflict of Interest

The authors declare that the research was conducted in the absence of any commercial or financial relationships that could be construed as a potential conflict of interest.
